# Organization of Neuropeptide Y-Immunoreactive Cells in the Mongolian gerbil (*Meriones unguiculatus*) Visual Cortex

**DOI:** 10.3390/cells10020311

**Published:** 2021-02-03

**Authors:** Myung-Jun Lee, Won-Tae Lee, Chang-Jin Jeon

**Affiliations:** Department of Biology, School of Life Sciences, BK21 FOUR KNU Creative BioResearch Group, College of Natural Sciences, and Brain Science and Engineering Institute, Kyungpook National University, Daegu 41566, Korea; bridlee900@gmail.com (M.-J.L.); lwt9274@naver.com (W.-T.L.)

**Keywords:** visual cortex, neuropeptide Y, calcium-binding protein, Mongolian gerbil, immunocytochemistry

## Abstract

Neuropeptide Y (NPY) is found throughout the central nervous system where it appears to be involved in the regulation of a wide range of physiological effects. The Mongolian gerbil, a member of the rodent family Muridae, is a diurnal animal and has been widely used in various aspects of biomedical research. This study was conducted to investigate the organization of NPY-immunoreactive (IR) neurons in the gerbil visual cortex using NPY immunocytochemistry. The highest density of NPY-IR neurons was located in layer V (50.58%). The major type of NPY-IR neuron was a multipolar round/oval cell type (44.57%). Double-color immunofluorescence revealed that 89.55% and 89.95% of NPY-IR neurons contained gamma-aminobutyric acid (GABA) or somatostatin, respectively. Several processes of the NPY-IR neurons surrounded GABAergic interneurons. Although 30.81% of the NPY-IR neurons contained calretinin, NPY and calbindin-D28K-IR neurons were co-expressed rarely (3.75%) and NPY did not co-express parvalbumin. Triple-color immunofluorescence with anti-GluR2 or CaMKII antibodies suggested that some non-GABAergic NPY-IR neurons may make excitatory synaptic contacts. This study indicates that NPY-IR neurons have a notable architecture and are unique subpopulations of the interneurons of the gerbil visual cortex, which could provide additional valuable data for elucidating the role of NPY in the visual process in diurnal animals.

## 1. Introduction

Neuropeptide Y (NPY), a 36 amino acid polypeptide, was first discovered in the porcine hypothalamus and has been found to be one of the most abundantly expressed neuropeptides in the mammalian central and peripheral nervous systems [1,2,3]. Studies have reported the role of NPY in widespread physiological control mechanisms such as feeding [4,5,6,7], neurogenesis [4,5,8], memory [4,9,10,11], stress regulation [7,11,12,13,14], circadian rhythms [7,15,16], and pituitary hormone release [17,18]. Furthermore, the pathological relationship between NPY and various diseases, such as obesity [19,20,21], neurodegenerative disorders [8,12,22], cardiovascular disorders [23,24,25], gastrointestinal disorders [26], and renal diseases [27,28], has been reported.

NPY has been localized in the visual cortex of various animals. In the visual cortices of humans [29,30], monkeys [31,32,33,34], cats [35,36,37], rats [31,38,39], and mice [40], NPY-immunoreactive (IR) neurons were found to be primarily distributed in deep cortical areas. The majority of NPY-IR neurons in the mammalian visual cortex are non-pyramidal multipolar or bipolar cell types. NPY has also been found in non-mammalian species, such as the pigeon visual wulst (an avian equivalent of the mammalian striate cortex) [41,42], chameleon [43], frog [44], killifish optic tecta [45], and octopus optic lobe [46]. NPY-containing interneurons nearly exclusively express GABA [47,48,49]. However, there are variations in the cortical distributions, morphological types, and GABA expression in NPY-containing neurons among different species [29,30,31,32,33,34,36,37,38,39,40].

Calcium-binding proteins (CBPs) are thought to play a crucial role in calcium-mediated cell signaling pathways. Impaired regulation of calcium by CBPs disturbs cellular homeostasis, which can lead to the development of various neurodegenerative diseases [50,51,52,53]. Although the precise neuronal function of CBPs in neurons remains unclear, they have been successfully applied as valuable selective markers for distinguishing different neuronal subpopulations in the central nervous system. Of the numerous types of CBPs, the three EF-hand calcium-binding proteins, calbindin-D28K, calretinin, and parvalbumin, have been found in abundance in various neuronal subpopulations in the central nervous system [50,54]. Although there are species-specific variations, these proteins are distributed in distinct neuronal subpopulations in various mammalian visual cortices, including those in rodents [40,55,56,57].

Gerbils, small mammals of the family Muridae in the order Rodentia, have several unique physiological, anatomical, and behavioral characteristics that make them an appropriate laboratory model species [58]. Gerbils have been widely used as experimental models in various biomedical research areas such as stroke, parasitic diseases, viral diseases, and bacterial diseases [58,59,60,61,62]. In neuroscience research, gerbils have been used extensively in various areas such as brain development, behavior, and sensory systems [63,64,65]. Mice, which belong to the same family as gerbils, are the most commonly used vertebrate species for biomedical research, especially because they are the mainstay of transgenic technology. Although mice and gerbils both belong to the Muridae family, gerbils are physiologically, anatomically, and behaviorally different from mice. For studies on the central visual system, gerbils are more advantageous than mice because mice and rats are nocturnal animals with rod-dominated retina, whereas gerbils are diurnal animals with a higher proportion of cones to rods than that in mice and rats [63,66]. Some gerbils also have a specialized retinal region that is similar to the macular region of the primate, which is not present in mice and rats [67,68]. Therefore, gerbils have been widely used in studies of the central visual system [63,64,65,69,70,71,72,73].

NPY-IR neuronal architecture is well studied in rodents, such as mice [40] and rats [31,38,39]. Although gerbils constitute an excellent animal model for central visual system research, one of the most widely expressed neuropeptides in the brain, i.e., NPY, has not been localized in the gerbil visual cortex. Therefore, the purpose of the present study was to reveal the organization of NPY-IR neurons in the gerbil visual cortex, through immunocytochemistry, quantitative analysis, and conventional/confocal microscopy. In the visual cortex of various animals including mice, NPY-IR neurons are almost exclusively GABAergic interneurons. To understand whether there are any species-specific differences, we examined the expression patterns of GABA in NPY-IR neurons. Although calbindin-D28K, calretinin, and parvalbumin are largely localized in different types of cortical neurons, it is not known whether NPY-IR neurons in the gerbil visual cortex contain these CBPs. Therefore, we also determined whether calbindin-D28K, calretinin, or parvalbumin were specifically expressed in NPY-IR neurons. Finally, we investigated the possible existence of excitatory connections in non-GABAergic NPY-IR neurons by labeling excitatory synaptic markers, AMPA receptor GluR2 subunit (GluR2), and Ca^2+^/calmodulin-dependent protein kinase II (CaMKII).

## 2. Materials and Methods

### 2.1. Animals and Tissue Preparation

Twelve Mongolian gerbils (*Meriones unguiculatus*) (aged 3–4 months, weighing 70–90 g) obtained from a local vendor were used in this study. The animals were anesthetized using a mixture of ketamine hydrochloride (30–40 mg/kg) and xylazine (3–6 mg/kg). All animals were perfused intracardially with 4% paraformaldehyde and 0.3–0.5% glutaraldehyde in 0.1 M sodium phosphate buffer (pH 7.4) containing 0.002% calcium chloride. Following a pre-rinse with approximately 20 mL of phosphate-buffered saline (PBS, pH 7.4) over a period of 3 min, each gerbil was perfused with 100 mL of fixative for 10–15 min through a syringe needle inserted through the left ventricle and aorta. The animal was decapitated and the brain was removed from the skull. The brain was then placed in a fixative overnight and stored overnight in 0.1 M phosphate buffer (pH 7.4) containing 8% sucrose and 0.002% CaCl_2_. Then, the brain was mounted onto a chuck and cut into 50 μm thick coronal sections using a Vibratome 3000 Plus Sectioning System (Vibratome, Saint Louis, MO, USA). Guide for the Care and Use of Laboratory Animals (https://grants.nih.gov/grants/olaw/guide-for-the-care-and-use-of-laboratory-animals.pdf) was followed.

### 2.2. Horseradish Peroxidase Staining

Polyclonal rabbit anti-NPY (Immunostar, Hudson, WI, USA) was used as the primary antibody. The primary antibody was diluted 1:500 and the biotinylated secondary antibody was diluted 1:200. Standard immunocytochemical techniques and methods were used, as described previously [74,75]. As a negative control, some sections were incubated in the same solution without the addition of the primary antibody, and these control tissues exhibited no NPY immunoreactivity. The sections were examined and photographed on a Zeiss Axioplan microscope (Carl Zeiss Meditec Inc., Jena, Germany) with conventional or differential interference contrast (DIC) optics.

### 2.3. Fluorescence Immunohistochemistry

To double-label the sections for both NPY with GABA, CBPs, or somatostatin, and to triple-label the sections for NPY with GABA and calbindin-D28K, GluR2, or CaMKII, standard immunohistochemical methods were used, as described earlier. The primary antibodies used were rabbit anti-NPY (Immunostar), mouse anti-GABA (Sigma-Aldrich, Saint Louis, MO, USA), guinea pig anti-GABA (Sigma-Aldrich), rat anti-somatostatin (Millipore, Burlington, MA, USA), mouse anti-calbindin-D28K (Sigma-Aldrich), guinea pig anti-calbindin-D28K (Synaptic systems, Göttingen, Germany), mouse anti-calretinin (Millipore), mouse anti-parvalbumin (Sigma-Aldrich), mouse anti-GluR2 (Sigma-Aldrich), and mouse anti-CaMKII (Abcam, Cambridge, UK). The primary antibodies were diluted at 1:500 (NPY), 1:250 (GluR2), or 1:200 (GABA, CBPs, Somatostatin, and CaMKII). The secondary antibodies used were Cy3- or fluorescein (FITC)-conjugated anti-rabbit IgGs (Jackson ImmunoResearch Inc., Baltimore, PA, USA) for detecting NPY, Cy3-conjugated anti-rat IgG (Vector Laboratories, Burlingame, CA, USA) for detecting somatostatin, FITC-conjugated anti-mouse (Vector Laboratories) or Alexa fluor 647-conjugated anti-mouse IgGs (Thermo Fisher Scientific, Waltham, MA, USA) for detecting GABA, FITC-conjugated anti-mouse (Vector Laboratories) or guinea pig (Jackson ImmunoResearch Inc.) IgGs for detecting calbindin-D28K, FITC-conjugated anti-mouse IgG (Vector Laboratories) for detecting calretinin or parvalbumin, or Alexa fluor 647-conjugated anti-mouse IgG (Thermo Fisher Scientific, Waltham, MA, USA) for detecting GluR2 or CaMKII. The secondary antibodies were diluted at 1:200. Labeled sections were preserved under coverslips in Vectashield mounting medium (Vector Laboratories).

### 2.4. Quantitative Analysis

For the quantitative analysis of laminar distribution, the NPY-IR neurons were photographed using a Zeiss Axioplan microscope with a 20× objective. A total of 30 sections were sampled, each with a width of 2000 µm, from each of three animals (10 tissue sections from each animal). The number of labeled neurons was expressed as an average of cell numbers and as a percentage of the total population of labeled neurons. The percentage frequency was calculated as follows: NPY-IR neurons at each layer/NPY-IR neurons in total layers. In three animals, the morphological types and the average diameter and area of NPY-IR neurons were analyzed. The morphological types were determined for 175 neurons analyzed from 59 sections in three gerbils, and the average diameter and area of NPY-IR neurons were determined for 111 neurons analyzed from 22 sections in three gerbils. All analyses were conducted using a 40× Zeiss Plan-Apochromat objective. To obtain the best images, we analyzed the cells under DIC optics. Only the cell profiles containing a nucleus and at least one faintly visible nucleolus were included in the analysis. The average diameter and area of labeled neurons were computed using a digital camera (Carl Zeiss Meditec Inc.). A cursor was moved manually around the outer contour of each cell using the Zeiss AxioVison system. Images were adjusted according to brightness and contrast using the Adobe Photoshop CS software (Adobe Systems Inc., San Jose, CA, USA). Double-labeled neurons were counted from a total of 12 (except calbindin-D28K: total 18 sections) different tissue sections from each of the three animals, each 2000 µm in width, across all layers. Double- and triple-labeled images were obtained and viewed under a Zeiss LSM800 laser scanning confocal microscope (Carl Zeiss Meditec Inc.) with 40× and 100× objectives.

Drawings of the NPY-IR neurons were produced using a Zeiss Axioplan microscope (Carl Zeiss Meditec Inc.) with a 40× and 63× Zeiss Plan-Apochromat objective (Carl Zeiss Meditec Inc.). NPY-IR neurons were imaged on a computer monitor, and cells were drawn on acetate sheets. The final images were drawn using Adobe Photoshop CS (Adobe Systems Inc.).

## 3. Results

### 3.1. Laminar Distribution of NPY-IR Neurons

**Figure 1:**
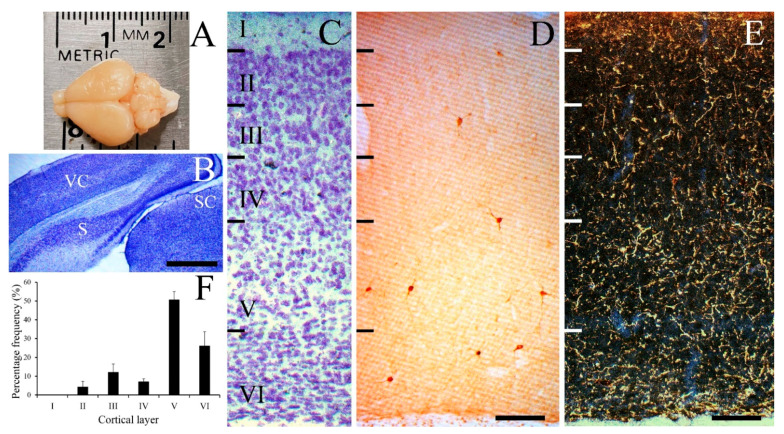
Laminar distribution of NPY-IR neurons with low-power photomicrographs in the gerbil visual cortex. Image of the gerbil brain in dorsal view (**A**) and low-power image of thionin stained gerbil visual cortex and surrounding areas (**B**). Thionin-stained section illustrating cortical lamination (**C**) and NPY-IR neurons (**D**). (**E**) Low-magnification dark-field photomicrograph of the gerbil visual cortex showing well-labeled NPY-IR fibers. (**F**) Histogram of the distribution of NPY-IR neurons in the gerbil visual cortex. NPY-IR neurons were mostly distributed in layers V and VI, but not in layer I. S, subiculum; SC, superior colliculus; VC, visual cortex. Scale bar = 1000 µm (**B**), and 100 µm (**D**,**E**).

The size of the Mongolian gerbil brain was approximately 2 cm in length (anterior to posterior) (Figure 1A). Figure 1B shows a low magnification image of the gerbil visual cortex in the coronal plane with thionin staining. NPY-IR neurons were sparsely and selectively distributed in the visual cortex of the gerbil. Figure 1C shows a thionin-stained section which reveals the cortical layers and laminar distribution of NPY-IR neurons (Figure 1D). NPY-IR neurons were located throughout all layers of the gerbil visual cortex except layer I. NPY-IR fibers were distributed throughout all layers of the visual cortex forming a plexus of labeled fibers with differential densities in different layers. Figure 1E, a dark field figure, is a representation of the distribution of the NPY-IR fibers in the gerbil visual cortex. The highest density of NPY-IR neurons was distributed in layer V. Layers II–IV showed considerably fewer numbers of NPY-IR neurons. Quantitative maps of the cells revealed the density of NPY-IR neurons in each layer (Figure 1F). Regarding the proportion of the total population of labeled neurons, 0% of NPY-IR neurons were found in layer I, 4.28% were found in layer II, 12.06% were found in layer III, 7.00% were found in layer IV, 50.58% were found in layer V, and 26.07% of NPY-IR neurons were found in layer VI.

### 3.2. Morphology of NPY-IR Neurons

**Figure 2:**
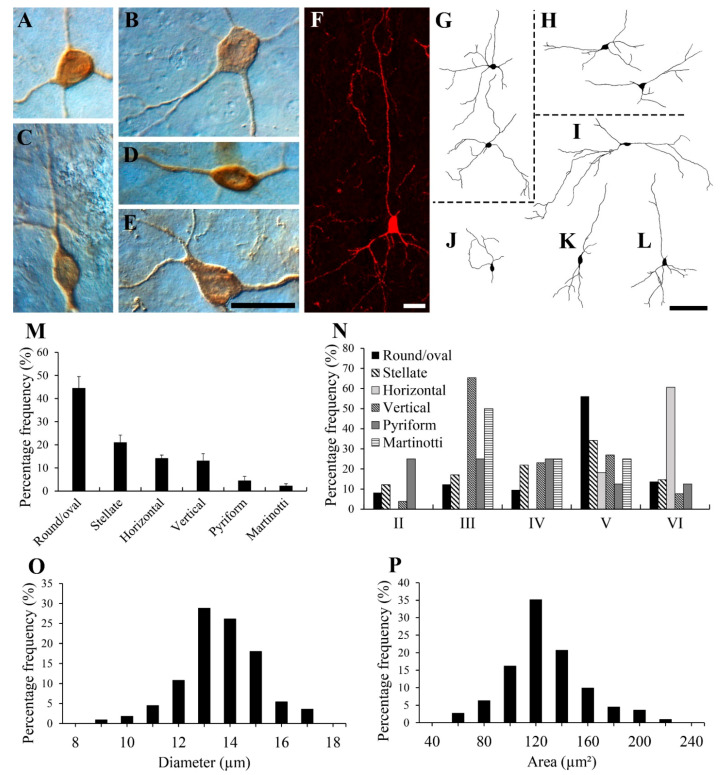
Types of NPY-IR neurons in the gerbil visual cortex. DIC photomicrographs (**A**–**E**), a fluorescence confocal photomicrograph (**F**), and drawings (**G**–**L**) of some NPY-IR neurons. (**A**,**G**) Multipolar round/oval type. (**B**,**H**) Multipolar stellate type. (**C**,**K**) Vertical fusiform type. (**D**,**I**) Horizontal type. (**E**,**J**) Pyriform type. (**F**,**L**) Martinotti cell. Although the drawings of the cells are from the best-labeled tissues, it is noteworthy that these representations of the NPY-IR neurons, as drawn here, are probably not realistic because of the possibility of a lack of complete filling of the cells due to the limitations of immunocytochemistry, and the truncation of some cell processes due to sectioning. Drawings have been simplified for the purpose of conveying the main features. (**M**) Histogram of the morphological distributions of NPY-IR neurons in the gerbil visual cortex. Round/oval neurons had the highest concentration of NPY-IR neurons, whereas the Martinotti cells comprised the smallest proportion. (**N**) Histogram of the layer distributions of different types of NPY-IR neurons in the gerbil visual cortex. The average diameter (**O**) and area (**P**) of 111 NPY-IR neurons in the gerbil visual cortex. The mean average diameter of the neurons was 13.13 µm (S.D. = 1.43) and the mean average area of the neurons was 137.52 μm^2^ (S.D. = 29.80). Scale bar = 20 µm (**E**,**F**), and 100 µm (**G**–**L**).

At least six types of NPY-IR neurons were found in the gerbil visual cortex as follows: multipolar round/oval, multipolar stellate, vertical fusiform, horizontal, pyriform, and Martinotti cells. Figure 2A–L show representative examples of each cell type. The large majority of NPY-IR neurons were round/oval cells, which are shown in Figure 2A,G. The cells had a round/oval-shaped cell body and multiple dendrites coursing in all directions. The next most common NPY-IR neurons were stellate cells, as shown in Figure 2B,H. Stellate cells had a polygonal-shaped cell body and multiple dendrites coursing in all directions. The multipolar round/oval and stellate cells had medium dendritic fields (200–300 µm in diameter). These cell types typically had 3–6 primary processes with sparsely branched processes. In general, the cell bodies of the round/oval cells were smaller than those of the stellate cells.

Figure 2C,K show a vertical fusiform cell with a vertical fusiform cell body, a primary long process ascending toward the pial surface, and a descending process. Figure 2D,I show a horizontal cell with a horizontal fusiform cell body and horizontally oriented processes. The fusiform cells had medium-to-large dendritic fields (300–400 µm in diameter) with two processes. In general, cell bodies of fusiform cells were also relatively smaller compared with those of the stellate cells. In the present study, the processes of the round/oval, stellate, and fusiform cells were aspinous.

Figure 2E,J show a pyriform cell with a pear-shaped cell body and a thick, proximal dendritic stump directed superficially toward the pial surface with a bouquet of dendrites. Figure 2F,L show a Martinotti cell. The Martinotti cells had small polygonal cell bodies. These cells showed very few short descending processes, and one apical process which extended toward the pial surface in layer I. Neuroglialform interneurons which have distinctive short multiple dendrites that spread in all directions, from small, round cell bodies, were not identified as NPY-IR neurons in the present study.

Figure 2M shows a histogram of the percentage of each cell type. Quantitatively, 44.57% ± 4.95% (mean ± S.D.) (78 of 175 cells) of NPY-IR neurons were round/oval, 21.14% ± 3.05% (37 of 175 cells) were stellate, 14.29% ± 1.29% (25 of 175 cells) were horizontal, 13.14% ± 3.04% (23 of 175 cells) were vertical fusiform, 4.57% ± 1.78% (8 of 175 cells) were pyriform, and 2.29% ± 0.89% (4 of 175 cells) were Martinotti cells.

Figure 2N shows a histogram of the frequency distributions of each cell type in each layer. The highest number of round/oval (56.16%) and stellate (34.15%) neurons were located in layer V. Vertical fusiform neurons were mostly distributed in layer III (65.38%), whereas horizontal neurons were mostly distributed in layer VI (60.61%). Pyriform neurons were distributed in layers II–VI and Martinotti cells were distributed in layers III–V in the present study. Pyriform and Martinotti cells were not present in sufficient numbers to draw a meaningful conclusion.

The average diameter and area of NPY-IR neurons in the gerbil visual cortex are shown in Figure 2O,P, respectively. The average diameter of 111 NPY-IR neurons measured in 22 sections from three animals ranged from 8.81 to 16.83 µm, with a mean of 13.13 µm (S.D. = 1.43 µm). The vast majority (90.99%, 101 of 111 cells) were smaller than 15.00 µm. There were no NPY-IR neurons with a diameter >18 µm in the gerbil visual cortex. The area of these cells ranged from 75.90 to 207.53 µm^2^, with a mean of 137.52 µm^2^ (S.D. = 29.80 µm^2^).

### 3.3. Colocalization of NPY with GABA, CBPs, Somatostatin, GluR2, or CaMKII

**Figure 3:**
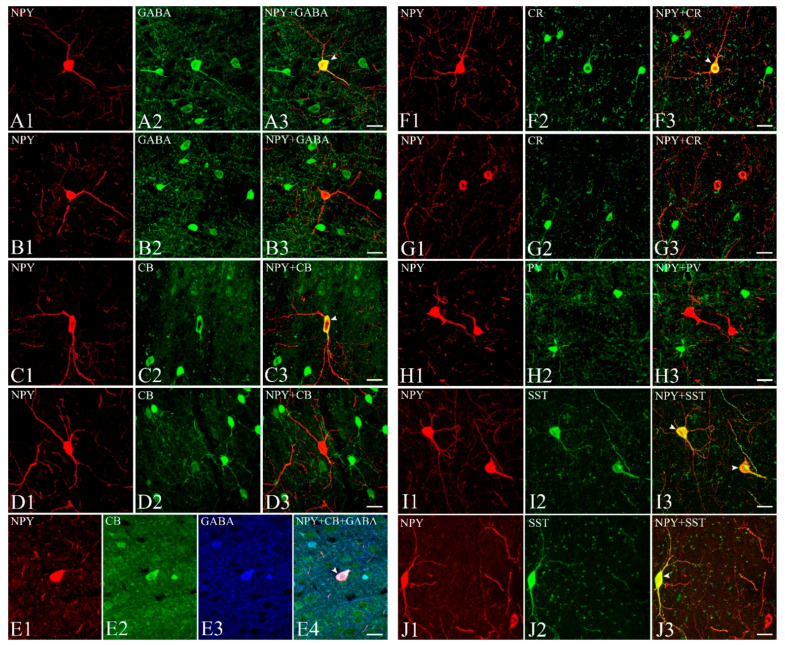
Colocalization of NPY with GABA-, CBPs-, or somatostatin-IR neurons in the gerbil visual cortex. Fluorescence confocal photomicrographs of the gerbil visual cortex immunostained for NPY (**A1**,**B1**,**C1**,**D1**,**E1**,**F1**,**G1**,**H1**,**I1**,**J1**), GABA (**A2**,**B2**,**E3**), calbindin-D28K (**C2**,**D2**,**E2**), calretinin (**F2**,**G2**), parvalbumin (**H2**), and somatostatin (**I2**,**J2**). Superimposed images of NPY with GABA (**A3**,**B3**), CBPs (**C3**,**D3**,**F3**,**G3**,**H3**), or somatostatin (**I3**,**J3**). Some of NPY-IR neurons were double-labeled with GABA (arrowhead in **A3**), calbindin-D28K (arrowhead in **C3**), calretinin (arrowhead in **F3**), and somatostatin (arrowheads in **I3**,**J3**). NPY-IR neurons did not colocalize with parvalbumin-IR neurons (**H3**). NPY-IR neurons double-labeled with calbindin-D28K were also labeled with GABA (**E4**). CB, calbindin-D28K; CR, calretinin; PV, parvalbumin; SST, somatostatin. Scale bar = 20 µm.

In the present study, we determined whether the NPY-IR neurons in the gerbil cortex colocalized with GABA, CBPs, or somatostatin. Figure 3 shows cells labeled with NPY (Figure 3(A1,B1,C1,D1,E1,F1,G1,H1,I1,J1)), GABA (Figure 3(A2,B2,E3)), CBPs (Figure 3(C2,D2,E2,F2,G2,H2)), or somatostatin (Figure 3(I2,J2)), and the superimposition of images of NPY with GABA (Figure 3(A3,B3)), CBPs (Figure 3(C3,D3,F3,G3,H3)), or somatostatin (Figure 3(I3,J3)), and NPY with calbindin-D28K and GABA (Figure 3(E4)). Most of NPY-IR neurons were co-labeled with GABA (arrowhead in Figure 3(A3)), but other cells were labeled with only one of the antibodies, i.e., NPY or GABA (Figure 3(B1–B3)). NPY-IR neurons were rarely labeled with calbindin-D28K (arrowhead in Figure 3(C1–C3)), whereas the majority of NPY-IR neurons did not co-express calbindin-D28K (Figure 3(D1–D3)). NPY-IR neurons labeled with calbindin-D28K expressed GABA (Figure 3(E4)). About one third of the NPY-IR neurons were labeled with calretinin (arrowhead in Figure 3(F3)), whereas many NPY-IR neurons did not co-express calretinin (Figure 3(G1–G3)). None of the NPY-IR neurons co-expressed parvalbumin (Figure 3(H1–H3)). Layer V contained the highest number of NPY-IR neurons double-labeled with calretinin. Quantitatively, 0% (0 of 92 cells) of NPY-IR neurons that were double-labeled with calretinin were found in layer I, 11.96% ± 3.20% (11 of 92 cells) were found in layer II, 20.65% ± 7.05% (19 of 92 cells) were found in layer III, 15.22% ± 2.81% (14 of 92 cells) were found in layer IV, 38.04% ± 5.76% (35 of 92 cells) were found in layer V, and 14.13% ± 4.23% (13 of 92 cells) of NPY-IR neurons were found in layer VI. There was no layer-specific distribution of NPY-IR neurons that were double-labeled with calbindin-D28K. Most of NPY-IR neurons were labeled with somatostatin (arrowheads in Figure 3(I3,J3)), but other cells were labeled with only one of the antibodies (Figure 3(J3)). No obvious relationship was detected between cell morphology and whether the cell was single-, double-, or triple-labeled. To estimate the percentage of double-labeled cells, we assessed each of the four sections from each of the three animals and counted the number of NPY-IR neurons and double-labeled cells across the layers of the visual cortex of the gerbil. As the number of double-labeled cells of NPY with calbindin-D28K was low, we assessed six sections from each of the three animals to increase the accuracy of statistical analyses. Quantitatively, 89.55% ± 8.58% (180 of 201 cells) of NPY-IR neurons were double-labeled with GABA, 3.75% ± 1.85% (10 of 267 cells) with calbindin-D28K, 30.81% ± 4.44% (61 of 198 cells) with calretinin, 0% (0 of 176 cells) with parvalbumin, and 89.95 ± 5.09% (224 of 249 cells) with somatostatin (Table 1).

**Figure 4:**
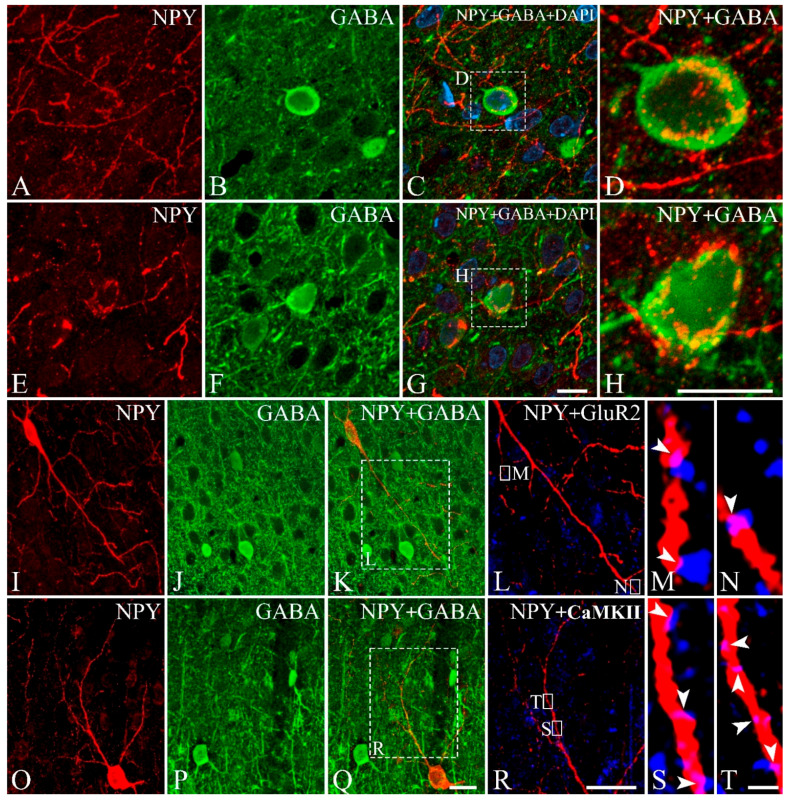
Double- and triple-labeling of NPY with GABA and GluR2 or CaMKII in the gerbil visual cortex. Expression of NPY (red, **A**,**E**,**I**,**O**), GABA (green, **B**,**F**,**J**,**P**), and GluR2 or CaMKII (blue represents the false color image of infrared fluorescence) in gerbil visual cortex. (**C**,**G**) Merged images of fluorescence NPY-IR fibers and GABA-IR neurons with 4′,6-diamidino-2-phenylindole (DAPI) staining. (**D**,**H**) Magnified images of boxes in C and G showing NPY-IR fibers surrounding the somata of GABA-IR neurons. (**K**,**Q**) Merged images of NPY-IR neurons which are not GABA-IR neurons. Magnified images of boxes in K and Q showing localization of NPY-IR fibers and GluR2- (**L**) or CaMKII-IR puncta (**R**). (**M**,**N**,**S**,**T**) Highly magnified images of boxes in L and R. NPY-IR fibers show colocalization (arrowheads, light pink) with GluR2- (**M**,**N**) or CaMKII-IR immunopuncta (**S**,**T**). A set of images NPY-IR fibers with GluR2- or CaMKII-IR immunopuncta was taken at single focal plane from the same field. Scale bar = 10 µm (**G**,**H**), 20 µm (**Q**,**R**), 1 µm (**T**).

Figure 4A–H shows the GABAergic interneurons (Figure 4B,F) that were surrounded by NPY-IR fibers (Figure 4A,C,E,G). Some NPY-IR fibers densely surrounded the GABAergic interneurons (Figure 4D,H). Figure 4I–T show triple-labeling with antibodies against NPY (Figure 4I,O), GABA (Figure 4J,P), and GluR2 (Figure 4L) or CaMKII (Figure 4R). Figure 4M,N,S,T show the processes of NPY-IR neurons that were not labeled with GABA (Figure 4K,Q) making contact with immunopuncta of GluR2 or CaMKII. When NPY and GluR2 or CaMKII were colocalized in the same focal plane, they were regarded as contacts. (arrowheads in Figure 4M,N,S,T).

## 4. Discussion

The present study showed that NPY-IR neurons in the gerbil visual cortex are distributed throughout layers II–VI, with the highest density detected in layer V. The labeled cells had various morphologies. NPY-IR neurons contained GABA, calbindin-D28K or calretinin at distinctively different ratios, whereas none of the NPY-IR neurons contained parvalbumin.

The distribution of NPY-IR neurons was primarily concentrated in the deep cortical layers of the gerbil visual cortex. The infragranular layers, layers V and VI, were the prominent location of NPY-IR neurons with the highest density in layer V. On the other hand, a relatively small number of NPY-IR neurons was distributed in other layers. Notably, there were no NPY-IR neurons in layer I of the gerbil visual cortex. Similar to the results of the present study, in humans [30] and monkeys [76], NPY-IR neurons have been found to be most frequently distributed in layer V or VI and more sparsely in supragranular layers. In cats, NPY-IR neurons have also been shown to be primarily distributed in infragranular layers and few in layers II/III [36]. In rats, the majority of NPY-IR neurons have been reported to be in deep cortical layers such as layer VI [39,77,78], similar to the results of the present study. Moreover, in cats [35,36] and rats [39,77,78], NPY-IR neurons were absent in layer I in adults, except some transient expression of NPY in layer I during development. However, there are subtle species differences in NPY expression. Berman and Fredrickson [29] demonstrated the highest number of NPY-IR neurons in layers II–III in the human visual cortex among the six cortical layers. Moreover, there were some NPY-IR neurons that were distributed in layer I in the visual cortices of humans [29,30,79] and macaques [33,34]. It is also notable that some differences in NPY distribution have been reported even within the same members of Muridae family. In contrast to the gerbil visual cortex in the present study, the mouse visual cortex was found to have a high distribution of NPY-IR neurons in layers II–III in addition to layer VI [40]. Some NPY-IR neurons were also found in layer I of the mouse visual cortex [40]. Thus, with respect to the distribution within the same members of the Muridae family, at least one solid conclusion can be drawn by comparing nocturnal mouse and diurnal gerbil data. Differences in the pattern of distribution of NPY-IR neurons in the two species are clearly seen. The functional significance of such species differences in NPY expression patterns remains to be elucidated. However, these differences in expression in layers may contribute to slight functional variations in different species.

The NPY-IR neurons in the gerbil visual cortex were morphologically diverse subpopulations of non-pyramidal neurons. Most were multipolar round/oval cells. Some vertical fusiform, multipolar stellate, pyriform, and horizontal neurons also expressed NPY. Consistent with the present result, NPY-IR neurons that were almost exclusively non-pyramidal cells have been found in humans [29,30,79], monkeys [31,33,34,76,80], cats [36,49], and mice [40], and a large proportion of the labeled cells were round/oval in shape. Oval cells were also the predominant type of NPY-IR neurons even in the invertebrate octopus optic lobe [46,81]. The combined results of the present and previous studies indicate that NPY-IR neurons are interneurons in the visual cortex. However, a few studies have demonstrated the existence of a few NPY-IR pyramidal cells in the human visual cortex [30,79]. These results suggest that some NPY-IR neurons may be projection neurons. A recent study demonstrated that NPY-IR labeled projection neurons in the inferior colliculus [82].

In the present study, we estimated the cell size of the NPY-IR neurons and found that the majority of NPY-IR neurons in the gerbil visual cortex were small to medium in size. However, in studies of other species, the size of NPY-IR neurons in the visual cortex has been shown to vary. Most of the NPY-IR neurons in the adult cat were not small in size [35,36,37], and large NPY-IR neurons measuring >20 µm in size were also found in cats [49]. In the human visual cortex, the NPY-IR neurons were highly varied in size (7–23 µm), with larger cells reported as well [29]. The invertebrate octopus also had a highly variable size (9–27 µm) of NPY-IR neurons in the optic lobe [46]. Although Gonchar et al. [40] did not measure NPY-IR neuron size, all the NPY-IR neurons in their study (Figures 1, 4 and 5) were small in size. There has been no report on the quantitative analysis of the cell size of NPY-IR neurons in the visual cortex of rodents to compare with the gerbils in the present study. Considering the overall morphologically distinct subpopulations of NPY-IR neurons with variable sizes among different species, NPY may serve varied biological functions in different species.

With respect to the double labeling data of NPY-IR neurons with GABA-IR neurons, we can also draw at least one solid conclusion within the same members of Muridae family. Differences in double labeling between mice and gerbils can be seen clearly. In the present study, we found that approximately 10% of NPY-IR neurons did not express GABA or somatostatin. In contrast to the gerbil visual cortex, almost all the NPY-IR neurons expressed GABA in the visual cortices of mice [40] and rats [39]. The NPY-IR neurons are supposed to be mostly GABAergic in the cerebral cortex [48,80,83,84], hippocampus [85], and hypothalamus [86] but not in the caudate-putamen nuclei [47]. A previous study also revealed that 97.7% of NPY-IR neurons expressed somatostatin in the human cortex [87]. These results suggest that a discrepancy exists in the GABA- and somatostatin-containing NPY-IR neurons among different areas and species. In our previous study of melanopsin-expressing intrinsically photosensitive retinal ganglion cells (ipRGCs), the M2 cells that have been frequently found in other rodents, we surprisingly did not find them in the Mongolian gerbil [69]. The present study on NPY and GABA and previous studies on ipRGCs indicate species-specific variation of neuronal subpopulations in mice and gerbils, both of which belong to the Muridae family.

The present study showed that NPY-IR neurons in the gerbil visual cortex were labeled by calretinin (30.81%) and calbindin-D28K (3.75%). However, none of the NPY-IR neurons in the gerbil visual cortex were labeled by parvalbumin. Similarly, NPY-IR neurons lacked parvalbumin in the cat [49,88] and mouse visual cortex [40]. In contrast to the findings of the present study, NPY-IR neurons in the cat visual cortex are not co-labeled with calbindin-D28K [49]. Some NPY-IR neurons in the mouse visual cortex have been reported to contain calretinin [40]. The present results show that all parvalbumin (100%), a majority of calbindin-D28K (96.25%), and over two-thirds of calretinin (69.19%) neurons in the gerbil visual cortex are distinct subpopulations that differ from those that co-express NPY. These results and the finding that approximately 10% of NPY-IR neurons did not co-localize GABA and somatostatin suggest diversification of neuronal cell types in the gerbil visual cortex. Thirteen distinct GABAergic neurons have been found in the mouse visual cortex based on the expression of CBPs and neuropeptides [40]. Moreover, Masland [89] suggested that there could be 1000 different cell types in the cortex. Markram et al. [90] suggested that “the diversity of interneurons might be required to achieve a balance between inhibition and excitation in the neocortex”. Genome-wide analysis of gene expression studies will help to clarify the diversity and reveal subtype-specific roles of these neurons [91].

The precise effects of NPY on the excitation and inhibition of cortical neurons are not fully understood. NPY mediates inhibitory synaptic transmission by releasing GABA onto cortical pyramidal neurons [92,93]. Our observation of NPY-IR fibers surrounding GABAergic cells suggests that some NPY-IR neurons may form synaptic contacts with GABAergic inhibitory neurons. In the present study, we found that at least 10% of NPY-IR neurons were not GABAergic cells. The close apposition of the processes of non-GABAergic NPY-IR neurons and GluR2 or CaMKII may suggest that these NPY-IR neurons can affect excitatory synaptic activity. Previous studies have shown that NPY can affect inhibitory as well as excitatory synaptic activity [92]. Although previous studies have shown that the immunopuncta observed through the confocal microscope represent synaptic sites [94,95], true synapses can only be identified with certainty using electron microscopy; this raises the question of whether these are true synapses. Therefore, electron microscopic studies will be necessary to confirm the synaptic features observed in the present study.

NPY performs a wide range of significant modulatory functions in the brain [7,8,11,12]. NPY also appears to play important roles in the visual cortex. For instance, NPY-IR fibers and varicosities near blood vessels in the visual cortices of humans and cats play a role in blood flow regulation [29,30,35,96]. In the macaque monkey, NPY-IR neurons are primarily located outside of cytochrome oxidase patches, indicating that NPY-IR neurons may play a role in pattern perception and binocular processing [32,33]. However, in humans, no relationship with cytochrome oxidase patches has been found [29]. NPY is related to neurological diseases and exerts neuroprotective functions [97,98]. Therefore, the decreased number of NPY-positive cells in the adult mouse visual cortex was found to be related to animal model of autism [99]. However, the particular role of NPY in the visual cortex is still poorly understood and remains to be elucidated.

## 5. Conclusions

Our study demonstrated that NPY-IR neurons were mostly present in layers IV and V, and the highest density was found in layer V in the gerbil visual cortex, with no labeled cells in layer I. A large number of NPY-IR neurons were round/oval cells, although other types of cells were also present. Unlike many other mammalian visual cortices where NPY neurons are almost exclusively GABAergic, at least 10% of NPY-IR neurons were not GABAergic or somatostatinergic interneurons in the gerbil visual cortex. Many of the NPY-IR neurons were distinct subpopulations of interneurons, which are independent from calbindin-D28K, calretinin, or parvalbumin-containing interneurons. Some non-GABAergic NPY-IR neurons may form excitatory synaptic contacts. Our findings should contribute to a better understanding of the rodent visual system and may provide fundamental insights for further physiological studies.

## Figures and Tables

**Table 1:** Percentage of NPY-IR neurons, and neurons double-labeled with GABA, CBPs, or somatostatin in the gerbil visual cortex.

Antibodies	Animal	No. Sections	No. NPY Cells	No. Double	% Double (Mean ± S.D.)
GABA	#1	4	53	46	86.79 ± 5.62
	#2	4	84	77	91.67 ± 6.36
	#3	4	64	57	89.06 ± 10.83
GABA total		12	201	180	89.55 ± 8.58
Calbindin-D28K	#1	6	84	2	2.38 ± 2.73
	#2	6	85	5	5.88 ± 5.30
	#3	6	98	3	3.06 ± 5.08
Calbindin-D28K total		18	267	10	3.75 ± 1.85
Calretinin	#1	4	49	14	28.57 ± 12.88
	#2	4	79	28	35.44 ± 11.32
	#3	4	70	19	27.14 ± 6.72
Calretinin total		12	198	61	30.81 ± 4.44
Parvalbumin	#1	4	64	0	0
	#2	4	59	0	0
	#3	4	53	0	0
Parvalbumin total		12	176	0	0
	#1	4	83	71	85.54 ± 0.58
Somatostatin	#2	4	78	69	88.46 ± 9.70
	#3	4	88	84	95.45 ± 2.38
Somatostatin total		12	249	224	89.95 ± 5.09

## Data Availability

Any additional data will be available upon request to the corresponding author.
